# Research on the stability evaluation method of anchored slopes based on group decision making and matter element analysis

**DOI:** 10.1038/s41598-021-96157-2

**Published:** 2021-08-16

**Authors:** Peng Xia, Chunye Ying

**Affiliations:** Faculty of Engineering, China University of Geosciences, Wuhan, 430074 China

**Keywords:** Natural hazards, Engineering

## Abstract

This research is focused on the evaluation method of anchored slope stability, and an accurate evaluation method with a simple operation is proposed. Group decision theory and the analytic hierarchy process are used to determine the weight of each evaluation element, the correlation degree of each indicator is determined based on matter element analysis theory, and inverse hierarchical calculations are performed based on the obtained weight value and correlation degree to finally obtain the criteria layer correlation degree used for stability evaluation. The results show the following: (1) the evaluation method better integrates the effects of multiple factors on the stability of the anchored slope, and the evaluation results are accurate and consistent with the actual situation of the project; (2) the evaluation method can make full use of the experience of the expert group and effectively avoid the evaluation error caused by the subjective deviation of a single expert; (3) the group decision theory-entropy model was introduced to realize the quantitative evaluation of the reliability of expert scoring and effectively improve the efficiency of expert discussion; and (4) the evaluation result is intuitive, and the correlation degree obtained can not only reflect the stability grade of the anchored slope but also reflect the "distance" between the anchored slope and other stability grades.

## Introduction

China has a vast territory and a wide distribution of landslides and is one of many countries that are seriously affected by landslides worldwide. With the rapid development of China's economic construction, especially in the implementation of the strategic decision of the "Western Development", large-scale water conservancy and hydropower projects have been implemented in areas with complex geological conditions and frequent landslide geological disasters. These projects have provided great economic benefits but inevitably induced many geological disasters such as landslides. Experts have proposed a variety of treatment means^1^, including retaining walls^2^, soil nails^3^, planting grass on slope faces^4^, rock bolts and frame beams^4^, and anti‑slide piles^5^. Among them, anchorage engineering is one of the most important treatment means^6,7^. Therefore, a large number of anchoring slopes can form in the reservoir area of hydropower stations. The stability evaluation of anchorage slopes is related not only to the success or failure of hydropower projects but also to the safety of people's lives and property in reservoir areas.

At present, the most commonly used methods for slope stability evaluation include the rigid body limit equilibrium method^8–11^, numerical simulation method^12,13^ and strength reduction method^14^. In addition to these three commonly used analysis methods, some scholars have also used artificial intelligence (AI)^15–20^, Monte Carlo simulation^21^ and multiple regression modelling^22^, fuzzy comprehensive evaluation^23^, extreme learning machine processing^24,25^, analytic hierarchy processing (AHP)^26,27^, grey relational analysis and reliability theory^28,29^ to analyse the stability of slopes. The evaluation methods of slope stability are summarized, as shown in Table 1 below.

**Table 1:** Slope stability evaluation method summary table.

Evaluation method of slope stability	Research content	References
Limit equilibrium method	A limit equilibrium analysis under the coupling effect of the shear dilatancy and strain softening is performed	Deng (2020)^8^
Limit equilibrium method	The factors of safety and critical slip surfaces obtained by the limit equilibrium method (LEM) and two finite element methods are compared	Liu et al. (2015)^9^
Limit equilibrium method	The failure mechanism and stability of active landslide are discussed	Iqbal et al. (2018)^10^
Limit equilibrium method	The influence of seepage variation on the slope stability is evaluated	Sun et al. (2013)^11^
Numerical simulation method	A numerical method is proposed and applied to evaluate slope stability under seismic loading	Lu et al. (2015)^12^
Numerical simulation method	Based on the three-dimensional distinct element code (3DEC), a new method for analysing the dynamic stability of rock slope wedge is proposed	Ni et al. (2014)^13^
Strength reduction method	This study presents a new nonlinear strength reduction method based on the Generalized Hoek–Brown criterion	Yuan et al. (2020)^14^
Artificial intelligence	An artificial neural network (ANN) model that can be employed for evaluating the factor of safety of Shiwalik Slopes in the Himalayan Region is developed	Ray et al. (2020)^15^
Artificial intelligence	Artificial neural network (ANN) and particle swarm optimization (PSO-ANN) models are established to predict the safety factor (FOS) of homogeneous slope, and a comparative analysis is carried out	Gordan et al. (2016)^16^
Artificial intelligence	An attempt is made to evaluate/predict SF of many homogenous slopes in static and dynamic conditions through applying various hybrid intelligent systems	Koopialipoor et al. (2019)^17^
Artificial intelligence	A hybrid stacking ensemble approach for enhancing the prediction of slope stability is proposed in this study	Kardani et al. (2021)^18^
Artificial intelligence	This study proposes a novel generalized artificial intelligence model for estimating the friction angle of clays from different areas/locations for evaluating slope stability	Zhang et al. (2021)^19^
Artificial intelligence	The study analyses and compares different artificial intelligence calculation models for slope stability analysis	Bharti et al. (2021)^20^
Monte carlo simulation	An attempt is made to evaluate/predict FOS of many homogenous slopes under different conditions using the Monte Carlo (MC) simulation technique	Mahdiyar et al. (2017)^21^
Multiple regression model	A multivariate regression model is established to estimate the slope safety factor under the influence of earthquake	Gordan et al. (2016)^22^
Fuzzy comprehensive evaluation	The application of fuzzy set theory in SMR classification is expounded. A Mamdani fuzzy algorithm for rock slope stability evaluation is established	Daftaribesheli et al. (2011)^23^
Extreme learning machine	A new regularized online sequence extreme learning machine is proposed, which combines variable forgetting factor (FOS-ELM) to predict slope stability	Deng et al. (2020)^24^
Extreme learning machine	This study presents slope stability evaluation and prediction with the approach of a fast robust neural network named the extreme learning machine	Liu et al. (2014)^25^
Analytic hierarchy process	The implementation of two semi-quantitative landslide evaluation methods, rock engineering system (RES) and analytic hierarchy process (AHP) is compared	Rozos et al. (2011)^26^
Analytic hierarchy process	The Analytic Hierarchy Process (AHP) is used to evaluate the landslide susceptibility of rock slopes	Suh et al. (2011)^27^
Reliability theory	This study develops an MCS-based approach for efficient evaluation of the system failure probability of slope stability in spatially variable soils	Jiang et al. (2014)^28^
Reliability theory	The universal transfer coefficient method popularized by Chinese standards is used as the performance function to evaluate the reliability of reservoir landslide	Bi et al. (2012)^29^

However, these methods still have some shortcomings. For example, the rigid body limit equilibrium method is based on a variety of assumptions, which leads to inconsistencies between the calculation conditions and the actual situation of the project, causing evaluation deviation^9,30^. Numerical simulation analysis requires a large amount of data support. However, slope stability analysis and design usually can only use datasets with limited densities, which leads to the conventional use of advanced numerical simulation programmes, such as FLAC, which are often impractical and not always necessary^31^. The weakness of the strength reduction method is that it cannot locate some locally slipping surfaces^32^. When the neural network is used for slope stability evaluation, it is difficult to reasonably determine the network structure; the training speed is slow, and the phenomenon of falling into the local minimum solution exists^33^. The penalty parameters of the support vector machine (SVM) method are robust and difficult to identify^34^. Artificial intelligence methods often require a large number of engineering cases for training, but actual engineering projects often do not have the conditions necessary to provide a large number of engineering cases.

As mentioned above, many scholars have applied different methods to the evaluation of slope stability and achieved good results. However, these methods still have some shortcomings. This paper introduces a new evaluation method for the stability of anchorage slopes, which combines the analytic hierarchy process, expert group discussion and matter-element analysis to evaluate the stability of the anchorage slope. The AHP and group eigenvalue method (GEM) are more practical decision-making methods. These two methods combine qualitative and quantitative methods to solve various decision problems and have the advantages of system flexibility and simplicity. They are widely used in different fields^35–40^. However, they each have their own disadvantages. For example, for the AHP, it is difficult to construct a consistent judgement matrix due to the diversity of targets and the inevitable mistakes people will make when making judgements. The GEM is too general and vague in its thinking^41^.

Matter element analysis theory^42,43^ is a discipline that studies the law and method of solving contradictory problems and can be used to formalize the process of solving problems. When the theory of matter-element analysis is applied to the problem of evaluation, it can combine quantitative and qualitative factors to obtain the comprehensive level of an evaluation indicator. It can reasonably obtain the internal structure of components as well as the relationship and changes taking place between them. Jia Chao^44^ summarized the advantages of matter-element analysis theory as follows.

1. The selected factors can be considered according to local conditions;
2. The type and number of factors to be considered are unrestricted;
3. The results of the investigation and experiment can be utilized to the maximum extent;
4. The incompatible problem can be turned into a compatible problem^45^.

Based on these advantages, matter-element analysis theory has been applied to water quality assessment^46,47^ development land applicability assessment^48^, tunnel karst water inrush risk assessment^49^, etc. In addition, Zhang^50^ applied the matter-element analysis theory to the stability analysis of mine slopes. Based on the above advantages and the research of scholars, matter-element analysis theory is applied to the stability evaluation of anchorage slopes in this paper.

As stated above, we can see the advantages of group decision-making theory and matter-element analysis theory, as well as some disadvantages of commonly used slope stability evaluation methods. However, few existing studies combine group decision theory and matter-element analysis theory to carry out slope stability evaluation. In this paper, a method for the stability evaluation of anchorage slopes is proposed, which combines group decision theory, AHP and matter-element analysis theory. AHP and GEM were combined to determine the weight of each evaluation indicator. This solution retains the scientific analysis process of AHP and avoids the problem of the inconsistency in the break matrix encountered in the solution of AHP. Group decision theory is introduced to make full use of the experience of the expert group. The evaluation error caused by individual knowledge differences and subjective judgement deviations can be effectively avoided. At the same time, the entropy model^51,52^ is introduced into the group discussion to realize the quantitative evaluation of the reliability of expert group decision making and improve the efficiency of discussion. Material element analysis theory is used to synthesize the influence of multiple factors on slope stability.

## Evaluation model

### Methods and procedure

Considering the characteristics of the anchorage slope, its stability is influenced not only by its factors, such as the geometric size of the slope, but also by the influence of the stability of the anchorage structure on the slope, such as the bearing capacity of the anchor solid. Therefore, this paper introduces the evaluation indicator and grading standard of the existing research results^53^. The implementation steps are as follows.

**Figure 1:**
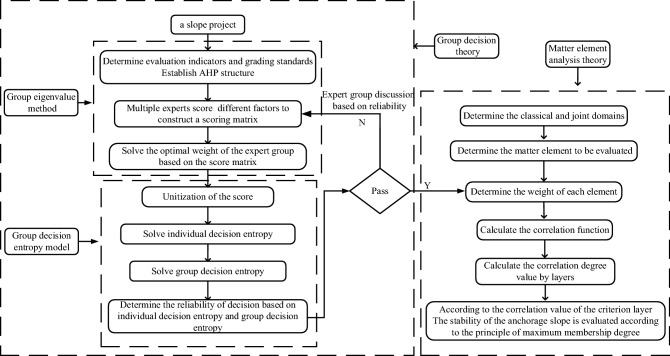
Flow chart of the anchor slope stability evaluation method based on group decision theory and matter element analysis theory.

1. Determine the stability evaluation indicator and classification standard of the anchorage slope;
2. Calculate the weights and expert reliability of each evaluation indicator based on group decision theory and the group decision entropy model;
3. Calculate the correlation degree based on matter element analysis theory;
4. Evaluate the stability of the anchorage slope;

The flow chart of the evaluation method is shown in Fig. 1.

### Evaluation indicator and grading standard

**Figure 2:**
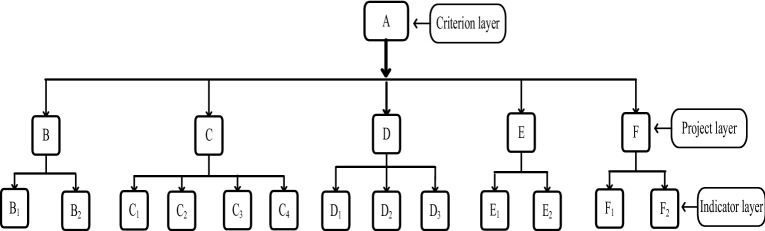
Hierarchical analysis structure of anchor slope stability evaluation.

This paper introduces existing research results^53^ and the indicators and grading standards used for the stability evaluation of anchoring slopes, including 13 indicators in five projects at three levels, which are of great value to the present research. The three levels are the criterion layer, project layer and indicator layer. The criterion layer has only one element, the stability of the anchor slope, denoted by A. The project layer consists of five projects, which are the geometric condition of the slope, geological structure and geologic process, anchoring body bearing capacity, hydrological climate and other factors and are represented, respectively, by B–F. There are 13 indicators in the indicator layer, and the slope angle and total slope height are represented by B1 and B2, respectively. The lithology, relationship between slope direction and stratum strike, internal friction angle and cohesion are represented by C1–C4, respectively. The bond strength between the cement-based grout and anchor hole wall, the bond strength between the rock bolt and cement-based grout, and the safety factor of the rock bolt are represented by D1–D3, respectively. Groundwater and process rainfall are, respectively, expressed by E1 and E2, and the maximum seismic intensity and human factor are, respectively, expressed by F1 and F2. The analytic hierarchy structure of the stability evaluation of the anchorage slope is shown in Fig. 2 below:

The qualitative indicators can be quantitatively processed according to existing research results^54^. The influence of each indicator on the stability of the anchorage slope can be divided into five grades: very favourable is 100 points, favourable is 85 points, general is 65 points, unfavourable is 30 points, and unfavourable is 10 points.

The influencing factors were as follows: no influence was 100 points, slight influence was 85 points, weak influence was 65 points, strong influence was 30 points, and strong influence was 10 points.

### Indicator weights and expert decision-making reliability

**Figure 3:**
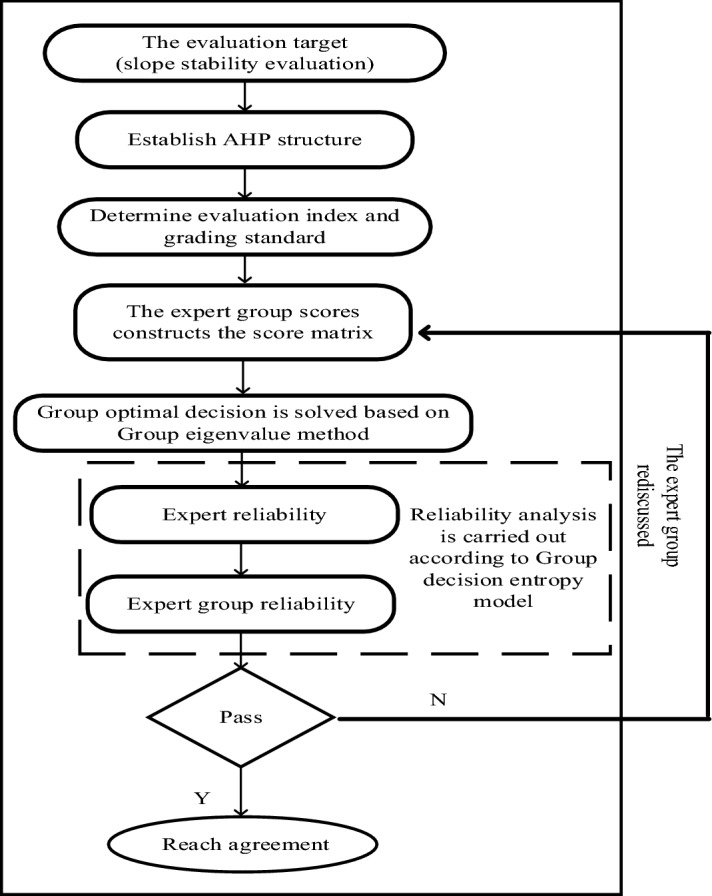
Group discussion change weight flowchart.

The differing importance of each indicator is manifested as different weight values. In this paper, group decision theory is used to determine the weight, and a group decision entropy model is used to calculate the reliability of expert decisions. The steps to solve the weight of each evaluation index and the reliability of expert decisions are as follows:

1. Construction of score matrix.
  Consisting of *S*_1_, *S*_2_,…, *S*_*m*_ is a group decision system *G* composed of m experts, and the evaluation objects are *B*_1_,* B*_2_,…, *B*_*n*_*.*
  The score of expert *S*_*i*_ on the evaluated indicator *B*_*j*_ is denoted as *x*_*ij*_(*i* = 1, 2,…, *m*; *j* = 1, 2,…, *n*)*.* The larger the value of *x*_*ij*_ is, the more important the target *B*_*j*_. The score order matrix *X* composed of the scores of group *G* is as follows:
  1 $$x = \left[ {\begin{array}{*{20}l} {x_{11} } \hfill & {x_{21} } \hfill & \cdots \hfill & {x_{1n} } \hfill \\ {x_{21} } \hfill & {x_{22} } \hfill & \cdots \hfill & {x_{2n} } \hfill \\ \vdots \hfill & \vdots \hfill & {} \hfill & \vdots \hfill \\ {x_{m1} } \hfill & {x_{m2} } \hfill & \cdots \hfill & {x_{mn} } \hfill \\ \end{array} } \right]$$
  where *x*_*mn*_ represents the score of expert *S*_*m*_ for evaluation subject *B*_*n*_. The higher the score of the indicator is, the more important the subject.
2. Solve the optimal weight.
  Assuming that expert S* with the highest decision-making level has the most accurate score (100% reliability); their score vector is as follows:
  $$x_{*} = \left( {x_{*1} ,x_{*2} , \ldots ,x_{*n} } \right)^{T}$$
  where S* is defined as the ideal expert, and S* is the expert with the highest consistency on the knowledge of the evaluated object and group *G*; that is, the decision conclusion of S* is completely consistent with that of *G*, and the difference between S* and the individual expert is the least.
  According to existing research results^55^, the square matrix **F** is constituted by the score matrix scored by group experts, where *X* * is the eigenvector corresponding to solving the maximum eigenroot of the square matrix **F**.
  $${\varvec{F}} = {\varvec{X}}^{T} \cdot{\varvec{X}}$$
  where ***X*** is the scoring matrix scored by the group expert.
  In this paper, the power method in numerical algebra is used to solve the eigenvector corresponding to the maximum eigenroot^40^. The specific solving steps are as follows:
  ① Let *k* = 0, ${\varvec{y}}_{{\varvec{0}}} = \left( {\frac{1}{n},\frac{1}{n}, \ldots ,\frac{1}{n}} \right)^{T}$, ${\varvec{y}}_{{\varvec{1}}} = {\varvec{F}}\cdot{\varvec{y}}_{{\varvec{0}}}$, ${\varvec{z}}_{{\varvec{1}}} = \frac{{{\varvec{y}}_{{\varvec{1}}} }}{{\left\| {{\varvec{y}}_{{\varvec{1}}} } \right\|_{2} }}$;where *n* is the number of evaluated schemes, and ***F*** is the square matrix obtained by multiplying the score matrix ***X*** and its inversion matrix.
  ② Let ${\varvec{y}}_{k + 1} = {\varvec{F}}\cdot{\varvec{z}}_{k}$ and ${\varvec{z}}_{k + 1} = \frac{{{\varvec{y}}_{k + 1} }}{{\left\| {{\varvec{y}}_{k + 1} } \right\|_{2} }}$ where $k = 1,2, \ldots$.
  ③ Let $\left| {{\varvec{z}}_{k} \to k + 1} \right|$ represent the largest absolute value of the difference between the components corresponding to ${\varvec{z}}_{k}$ and ${\varvec{z}}_{k + 1}$. When the accuracy requirement is $\varepsilon$, it is judged whether $\left| {{\varvec{z}}_{k} \to k + 1} \right|$ is smaller than $\varepsilon$. If $\left| {{\varvec{z}}_{k} \to k + 1} \right|$ is less than $\varepsilon$, then ${\varvec{z}}_{k + 1}$ is the requested ${\varvec{x}}_{*}$; otherwise, step ② is repeated to calculate ${\varvec{z}}_{k + 1}$ again until the accuracy requirement is reached.
  According to the optimal scoring vector ${\varvec{x}}_{*} = \left( {x_{*1} ,x_{*2} \ldots ,x_{*n} } \right)^{T}$, the indicator weight $q_{i}$ is solved as follows:
  $$q_{i} = {{x_{*i} } \mathord{\left/ {\vphantom {{x_{*i} } {(x_{*1} + x_{*2} + \cdots + x_{*n} )}}} \right. \kern-\nulldelimiterspace} {(x_{*1} + x_{*2} + \cdots + x_{*n} )}}$$
  where $q_{i}$ is the weight value of scheme i for the group decision and *n* is the total number of schemes.
3. Solve the expert decision level vector.
  ① Construct a normalized scoring matrix:
    2 $$\begin{aligned} & d_{ij} = {{x_{ij} } \mathord{\left/ {\vphantom {{x_{ij} } {\sqrt {x_{i1}^{2} + x_{i2}^{2} + \cdots + x_{in}^{2} } }}} \right. \kern-\nulldelimiterspace} {\sqrt {x_{i1}^{2} + x_{i2}^{2} + \cdots + x_{in}^{2} } }},\quad 0 \le d_{ij} \le 1 \\ & i = *,1,2, \ldots ,m;\quad j = 1,2, \ldots ,n \\ \end{aligned}$$
    3 $$\begin{aligned} & {\varvec{D}}_{{\varvec{i}}} = (d_{i1} ,d_{i2} , \ldots ,d_{in} )^{T} ,\quad i = *,1,2, \ldots ,m \\ & {\varvec{D}} = ({\varvec{D}}_{{\varvec{1}}} ,{\varvec{D}}_{{\varvec{2}}} , \ldots ,{\varvec{D}}_{{\varvec{m}}} )^{T} = (d_{ij} )_{m \times n} \\ \end{aligned}$$
    where* i* represents expert *i*, * represents the ideal expert assumed in “Indicator weights and expert decision-making reliability” of this paper, *j* represents scheme *j*, and *x*_*ij*_ represents the scoring value of expert* i* for scheme *j*. $d_{ij}$ represents an element of the normalized processed score matrix corresponding to *x*_*ij*_. ${\varvec{D}}_{{\varvec{i}}}$ represents the score of scheme *n* by expert *i* and the normalized score matrix. ${\varvec{D}}$ represents a normalized score matrix composed of m experts and n schemes.
  ② Calculate the expert decision level vector.
    We determine the expert-level decision vector ${\varvec{E}}_{{\varvec{i}}} = (e_{i1} ,e_{i2} , \ldots ,e_{in} )$ according to the normalized scoring matrix ***D***:
    4 $$\begin{aligned} & e_{ij} = 1 - \left| {N_{*J} - N_{ij} } \right| - \left| {d_{*j} - d_{ij} } \right| \\ & i = *,1,2, \ldots ,m\quad j = 1,2, \ldots ,n \\ \end{aligned}$$
    where $e_{ij}$ represents the component of the decision vector of level $E_{i}$ of expert $S_{i}$ with respect to scheme *j.* $N_{ij}$ represents the ranking of the evaluated programme $B_{1} ,B_{2} , \ldots ,B_{j}$ determined by the expert score $S_{i}$. The scheme with the highest score is taken as 1, and the scheme with the lowest score is taken as *j*.
4. Solve the reliability of expert decision.
  Decision entropy^51,52^ measures the expert's decision level with the inaccuracy or uncertainty of the expert's conclusion.
  It is equal to the sum of the generalized entropy of the horizontal components of the decision.
  The calculation method is as follows:
  5 $$H_{i} = \sum\limits_{j = 1}^{n} {h_{ij} }$$
  6 $$h_{ij} = \left\{ {\begin{array}{*{20}l} { - e_{ij} lne_{ij} } \hfill & {1/e \le e_{ij} \le 1} \hfill \\ {2/e - e_{ij} \left| {ine_{ij} } \right|} \hfill & {0 \le e_{ij} \le 1/e} \hfill \\ {2/e - e_{ij} \sqrt {ln^{2} ( - e_{ij} ) + \pi^{2} } } \hfill & { - 1 \le e_{ij} \le 0} \hfill \\ \end{array} } \right.$$
  7 $$H_{G} = \frac{1}{m}\sum\limits_{i = 1}^{m} {H_{i} }$$
  where $H_{i}$ represents the decision entropy of expert *i*. $e_{ij}$ represents the component of the decision vector of level ${\varvec{E}}_{{\varvec{i}}}$ of expert *i* with respect to scheme *j*. $H_{G}$ represents the decision entropy of the expert group. *m* represents the number of experts.
  Combining the decision entropy value and the "Decision Reliability and Decision Entropy Value Table", the reliability of expert decisions and group decisions is obtained^51^.
5. Group discussion of changing weights.
  The reliability of the whole expert group and each expert group is calculated based on the decision entropy model, and the weight scheme with low reliability is rediscussed to finally obtain the weight value with high reliability. The group discussion process is shown in Fig. 3 below:

### Calculation of correlation degree and stability evaluation

According to the relevant research results^56,57^, the calculation process of matter-element analysis theory divides the evaluation indexes into several grades, gives the grading standards for each grade, and calculates the evaluation indexes according to the corresponding grading standard set. The correlation degree value between the index and each level set is obtained. The larger the correlation degree is, the more consistent the index is with this level. In actual evaluation, the principle of maximum correlation degree is adopted, that is, the grade set corresponding to the value of maximum correlation degree is the grade determined by the final evaluation. The calculation process of the correlation degree is as follows:

1. Determine the classical domain.
  The classical domain is shown as follows:
  8 $$R_{0} = (P_{0} ,C,V_{0} ) = \left[ {\begin{array}{*{20}l} {P_{0} ,} \hfill & {C_{1} ,} \hfill & {X_{01} } \hfill \\ {} \hfill & {C_{2} ,} \hfill & {X_{02} } \hfill \\ {} \hfill & \cdots \hfill & \cdots \hfill \\ {} \hfill & {C_{n} ,} \hfill & {X_{0n} } \hfill \\ \end{array} } \right]$$
  where $P_{0}$ refers to the evaluation level. $C_{1} ,C_{1} , \ldots ,C_{n}$ are n different features, and in this evaluation method, these represent the elements of the indicator layer.
  $X_{01} ,X_{02} , \ldots ,X_{0n}$ is the range of values of $P_{0}$ with respect to $C_{1} ,C_{1} , \ldots ,C_{n}$, which is the classical domain.
2. Determine the joint domain
  9 $$R_{p} = (P,C,X_{P} ) = \left[ {\begin{array}{*{20}l} {P,} \hfill & {C_{1} ,} \hfill & {X_{p1} } \hfill \\ {} \hfill & {C_{2} ,} \hfill & {X_{p2} } \hfill \\ {} \hfill & { \cdots ,} \hfill & \cdots \hfill \\ {} \hfill & {C_{n} ,} \hfill & {X_{pn} } \hfill \\ \end{array} } \right]$$
  where $P$ refers to all evaluation levels, $X_{p1} ,X_{p2} , \ldots ,X_{pn}$ is $P$, and the value range of $C_{1} ,C_{1} , \ldots ,C_{n}$ is the joint domain.
3. Determine the matter element to be evaluated.
  The matter element to be evaluated is $C_{1} ,C_{1} , \ldots ,C_{n}$ in Eq. (8) and Eq. (9) and is the different characteristics of the evaluation level.
4. Determine the weight of each element.
  The weight of each element is determined by the group decision theory in “Indicator weights and expert decision-making reliability” above.
5. Calculate the correlation function.
  The measured values of the indicator layer elements of the AHP are substituted into the corresponding correlation degree function^9,30,42,43^ to obtain the correlation degree value for evaluation.
  10 $$K_{j} (x_{i} ) = \left\{ {\begin{array}{*{20}l} {\frac{{ - 2\rho (x_{i} ,x_{0ji} )}}{{\left| {x_{0ji} } \right|}}} \hfill & {(x_{i} \in x_{0ji} )} \hfill \\ {\frac{{\rho (x_{i} ,x_{0ji} )}}{{\rho (x_{i} ,x_{pj} ) - \rho (x_{i} ,x_{0ji} )}}} \hfill & {(x_{i} \notin x_{0ji} )} \hfill \\ \end{array} } \right.$$
  In the equation $K_{j} (x_{i} )$ is the correlation degree of evaluation indicator *i* to grade *j*, where *j* is level 1, 2, 3, 4, and 5; and $x_{i}$ is the measured value of evaluation indicator *i*;
  $x_{0ji}$ is the grading standard of evaluation indicator *i* relative to grade *j,* which is the classical domain;
  $x_{pj}$ is the magnitude value range of evaluation indicator *i* relative to grade *j*, which is the joint domain;
  $\rho (x_{i} ,x_{0ji} )$ is the distance between point $x_{i}$ and the finite real number interval $x_{0ji}$ = <a,b>, which is called the distance function.
  11 $$\rho (x_{i} ,x_{0ji} ) = \left\{ {\begin{array}{*{20}l} {a - x} \hfill & {\left( {{\text{x}} \le \frac{a + b}{2}} \right)} \hfill \\ {x - b} \hfill & {\left( {{\text{x}} \ge \frac{a + b}{2}} \right)} \hfill \\ \end{array} } \right.$$
6. Calculate the correlation degree value by layers.
  According to Eqs. (10)–(11) in “Calculation of correlation degree and stability evaluation”, the correlation degree values of each element in the indicator layer can be directly obtained. Then, according to the analytic hierarchy structure, the weighted sum of each element in the project layer and the criterion layer can be obtained successively.
  Based on the obtained correlation degree value, the stability of the anchorage slope is evaluated according to the maximum correlation principle. In other words, among the correlation degree values of the elements of the criterion layer with respect to each grade, the grade corresponding to the largest value is the stability grade of the anchored slope.

## Case study

An anchoring slope near a highway is selected as an engineering example to illustrate the rationality of the evaluation method.

### Engineering situation

The project case is drawn from existing research results^53^. The slope of a road is located in the hilly area of a low mountain landform. The slope inclines to the east, the slope is 20°–30°, the slope height is 45 m, and the horizontal excavation depth is 5.9 m. The slope rock mass is mainly composed of thick and strong weathered rock strata. The trend of the weak structural plane is opposite to that of the slope surface. Joints have developed here, and the slope top is covered with a layer of residual soil. The basic seismic intensity of the area where the slope is located is less than 6°, belonging to the weak seismic area, and the maximum possible acceleration is 0.08 g. The cohesiveness of the slope rock mass C = 65 kPa, φ = 25°, and groundwater is abundant. Groundwater mainly consists of pore water in loose deposits and bedrock fissure water, which is mainly replenished by infiltration by meteoric water. The area where the slope is located is rainy with abundant rainfall. The maximum process rainfall is 180 mm, and the total rainfall is 400 mm for 12 consecutive days. After heavy rain, slopes frequently suffer from small landslides or collapses. The slope is reinforced by a prestressed anchor cable, and the grouting strength grade is M30.

### Values of each evaluation indicator

According to the engineering overview and the existing research literature^40^, the calculation parameters of each indicator of the anchorage slope are obtained as shown in Table 2 below.

**Table 2:** Values of each element of the indicator layer.

B_1_	B_2_	C_1_	C_2_	C_3_	C_4_	D_1_	D_2_	D_3_	E_1_	E_2_	F_1_	F_2_
25	45	25	50	25	0.065	500	80	2.1	50	180	2	10

### Calculation of indicator weights and decision reliability

In the evaluation of this project, the reliability of expert decision-making was required to be at least 90%. Six experts in this field were invited to discuss and score the importance of the five elements of the project layer and their respective corresponding indicator layer for the first time.

Due to space constraints, this paper does not provide numerical values to list these six score matrices.

The calculation method of weight and reliability described in “Indicator weights and expert decision-making reliability” is adopted to obtain the optimal weight vector and the decision entropy and reliability of each expert and group, as shown in Table 3 below.

**Table 3:** Summary table of weight vector, decision entropy and reliability.

Expert	B		C		D		E		F		Element of the project layer	
	Decision entropy	Reliability	Decision entropy	Reliability	Decision entropy	Reliability	Decision entropy	Reliability	Decision entropy	Reliability	Decision entropy	Reliability
S1	0.0257	95	0.0934	95	0.0194	99	0.0488	95	0.0483	95	0.0396	99
S2	0.0625	95	0.1218	95	0.0520	95	0.0379	95	0.0250	95	0.0157	99
S3	0.0273	95	0.1269	95	0.0157	99	0.0432	95	0.1503	90	1.6129	50
S4	0.0716	95	2.8910	40	0.0703	95	0.0517	95	0.0378	95	1.6659	50
S5	0.1112	90	0.2257	90	2.8901	40	0.0209	95	0.1661	90	0.0572	95
S6	0.0346	95	0.0979	95	0.3553	85	0.0663	95	0.0173	99	1.5967	50
Expert group	0.0555	95	0.5928	80	0.5671	75	0.0448	95	0.0741	95	0.8313	80
Weight vectors	{0.3431, 0.6569}		{0.2490, 0.2146, 0.3499, 0.1865}		{0.4684, 0.3150, 0.2166}		{0.6187, 0.3813}		{0.7693, 0.2307}		{0.2598, 0.1428, 0.3283, 0.1298, 0.1392}	

In Table 3, B–F are the decisions of the element weight of the indicator layer corresponding to the geometric condition of the slope, geological structure, geologic process, anchoring body bearing capacity, hydrological climate and other factors, respectively, in the project layer. The element of the project layer represents a decision on the weight of the five elements in the project layer. The decision schemes that fail to meet reliability requirements are marked in red in Table 3.

According to the reliability in “Calculation of indicator weights and decision reliability”, the reliability of the geological structure and the influencing factors of the geological process, the bearing capacity of the anchor solid and the elements of the project do not meet the requirements of 90%. By observing the corresponding summary table, it can be concluded that the low reliability of the fourth expert in the influencing factors of geological structure and geological action led to the low reliability of the whole group’s decision-making ability, and the low reliability of the fifth and sixth experts in the influencing factors of anchor solid bearing capacity led to a similar low reliability. The scoring schemes of these experts were discussed in a targeted way and then re-graded after the discussion, which effectively improved the efficiency of the discussion. If the reliability of most experts was low, for example, in the weight decision of project layer elements in Table 3, the reliability of three experts was only 50, accounting for half of the total number of people. It is possible that the experts' thinking was divergent and far from a consensus, so a second discussion was necessary.

After the second discussion, the experts graded the geological structure and the influencing factors of geological processes, the influencing factors of anchorage bearing capacity and the importance of each element of the project layer twice and obtained the optimal weight vector based on the second discussion and the decision entropy and reliability of each expert and group.

As shown in Tables 4 and 5 below.

**Table 4:** Summary of optimal weight vectors based on secondary discussion.

Weight vectors	The weight vector value
W_1_	{0.3431, 0.6569}
W_2_	{0.2357, 0.2150, 0.3702, 0.1791}
W_3_	{0.2420, 0.3926, 0.3655}
W_4_	{0.6187, 0.3813}
W_5_	{0.7693, 0.2307}
W_6_	{0.2723, 0.1388, 0.3244, 0.1277, 0.1388}

**Table 5:** Summary table of decision entropy and reliability based on the second discussion.

Expert	C		D		Element of the project layer	
	Decision entropy	Reliability	Decision entropy	Reliability	Decision entropy	Reliability
S1	0.0356	99	0.0254	99	0.0621	95
S2	0.0646	95	0.0177	99	0.0656	95
S3	0.0662	95	0.0326	95	0.0656	95
S4	0.0718	95	0.0418	95	0.0656	95
S5	0.0528	95	0.0355	95	0.0621	95
S6	0.0375	99	0.0297	95	0.0621	95
Expert group	0.0548	95	0.0304	95	0.0638	95

In Table 5, C–D represent the decisions of the weight of elements in the indicator layer corresponding to the influencing factors of geological structure and geological action and the bearing capacity of the anchor solid.

The project layer element represents a decision on the weight of the five elements in the project layer.

It can be seen from the calculation results that the experts gradually unified their ideas after discussion and had a more consistent understanding of the evaluation indicators. The reliability of the decision has reached more than 90%, meeting the predetermined reliability requirements.

Therefore, the optimal weight vector based on quadratic discussion, as shown in Table 4, is adopted in this paper as the weight value adopted in the evaluation of this project instance.

### Calculation of correlation degree value

Based on the solving method of the correlation degree described in “Calculation of correlation degree and stability evaluation”, the correlation degree of each indicator is calculated. The calculation results are shown in Table 6 below.

**Table 6:** Correlation degree of each element of the indicator layer.

Level	Indicators												
	B1	B2	C1	C2	C3	C4	D1	D2	D3	E1	E2	F1	F2
Level 1	− 0.17	− 0.25	− 0.71	− 0.41	− 0.33	− 0.74	− 0.44	− 0.20	− 0.10	− 0.41	− 0.52	0.67	0.67
Level 2	1.00	0.50	− 0.62	− 0.23	− 0.13	− 0.57	− 0.09	0.50	1.00	− 0.23	− 0.40	− 0.67	− 0.33
Level 3	− 0.17	− 0.10	− 0.17	0.86	0.86	− 0.35	0.59	− 0.43	− 0.10	0.86	− 0.20	− 0.75	− 0.71
Level 4	− 0.38	− 0.55	0.50	− 0.29	− 0.17	0.60	− 0.19	− 0.71	− 0.25	− 0.29	0.80	− 0.80	− 0.86
Level 5	− 0.50	− 0.78	− 0.38	− 0.44	− 0.35	− 0.19	− 0.39	− 0.78	− 0.36	− 0.44	− 0.14	− 0.82	− 0.89

Based on the optimal weight value of elements in each layer obtained in “Calculation of indicator weights and decision reliability” and the correlation degree value of elements in the indicator layer obtained in “Calculation of correlation degree value”.

The correlation degree values of elements of the project layer and the criterion layer are obtained as shown in Table 7 below.

**Table 7:** Correlation degree of each element of the project layer and the criterion layer.

Level	Indicators					
	B	C	D	E	F	A
Level 1	− 0.22	0.67	− 0.12	− 0.49	− 0.68	− 0.17
Level 2	− 0.51	− 0.34	0.40	0.10	− 0.35	0.19
Level 3	− 0.22	0.54	− 0.06	− 0.42	− 0.53	− 0.04
Level 4	− 0.45	− 0.30	0.45	0.13	− 0.33	− 0.35
Level 5	0.67	− 0.59	− 0.74	− 0.81	− 0.83	− 0.56

### Stability evaluation of anchorage slope

According to the correlation degree value of element A in the criterion layer in Table 7 and the principle of the maximum correlation degree, it can be concluded that the stability state of the anchorage slope evaluated in this paper is level 2, which is basically stable. The existing research results^53^ show that anchor slope engineering is in a relatively stable state and is consistent with the actual situation after anchor cable construction.

The evaluation results in this paper are consistent with the evaluation results of existing studies^53^ and the actual engineering situation. This shows that the evaluation method proposed in this paper is reasonable and feasible and can accurately evaluate the actual state of the anchorage slope.

Compared to the existing results^53^, the evaluation method proposed in this paper can fully absorb the experience of expert groups and effectively avoid the errors of evaluation results caused by the subjective differences of individual experts. The reliability of each expert decision and group decision can be intuitively obtained, the efficiency of expert discussion can be effectively improved, and the weight value of each element with high reliability can be obtained. The evaluation method can also obtain the correlation degree value between the anchorage slope and each grade, and the evaluation result is more intuitive, which is convenient for application in actual engineering sites.

## Discussion

In this paper, an anchoring slope near a highway is selected as an engineering example to illustrate the rationality of the evaluation method. However, for different types of anchorage slopes, the selected evaluation indicators will be different. For example, when the evaluation method is applied to the reservoir slope, it is necessary to add relevant indicators of reservoir water level change to the reconstructed index system. This paper mainly studies the evaluation methods, and the evaluation indicators, grading standards and engineering cases are all from the existing studies and references. In future research work, more systematic and accurate evaluation indicators and grading standards will be collected and sorted out, and more engineering cases will be used to verify the reliability of the evaluation method.

Compared with the limit equilibrium method and SRM method, the proposed method can quickly evaluate the stability of multiple anchoring slopes of the same type. For example, the stability of dozens of anchored slopes along highways needs to be assessed. The stability of multiple anchoring slopes can be evaluated quickly by establishing a set of evaluation index systems and calculating and analysing the corresponding indicators. For slopes with poor stability, the limit equilibrium method can be used to further evaluate the slope stability. Thus, the evaluation efficiency of the stability of the anchorage slope is effectively improved.

As mentioned in the Introduction, the AI method is widely used in slope stability evaluation; although it has many advantages, it still has some disadvantages. In particular, a large number of engineering examples are needed for training, and it is very time- and labour-consuming to obtain the stability state of these engineering examples in actual engineering practice. As mentioned above, this method has a high working efficiency when evaluating the stability of the anchorage slope and can quickly obtain the stability states of multiple slopes. It can provide strong support for the training of models in AI methods.

This method makes full use of the AHP to establish the analytic hierarchy structure when constructing the indicator system. Figure 2 shows the analytic hierarchy structure of the anchorage slope. In “Calculation of correlation degree and stability evaluation”, this hierarchy structure is also made full use of when calculating the correlation degree of the anchorage slope. In “Indicator weights and expert decision-making reliability”, when solving the optimal weight vector, GEM is adopted to solve the problem of the inconsistent judgement matrix in AHP and simplify the solution process. In addition, the entropy model is introduced into group decision making to conduct a quantitative evaluation of the reliability of expert decision making, as shown in Table 2. The decision entropy and reliability scores of each expert and group decision are obtained. In the second discussion, only the low reliability decision is discussed, which improves the efficiency of group decision making.

## Conclusions

On the basis of summarizing the commonly used evaluation methods of slope stability, group decision theory, analytic hierarchy process and matter-element analysis theory, this paper proposes an evaluation method of anchorage slope stability, which combines group decision theory, analytic hierarchy processing and matter-element analysis theory. Based on engineering example verification, the following conclusions are drawn.

1. The anchorage slope stability analysis method can make full use of the theory of matter-element analysis considering the influence of various factors of slope stability for anchoring and make full use of geological exploration data, meteorological and hydrological data, and anchor cable design data and results. The evaluation results are accurate and comply with the actual engineering situation.
2. The stability analysis method of anchorage slopes based on group decision theory can give full play to the experience of expert groups and effectively avoid the evaluation error caused by the subjective deviation of a single expert.
3. The group decision theory-entropy model is introduced to achieve the quantitative evaluation of the reliability of expert scoring, which can intuitively obtain the reliability of each expert and expert group decision, improve the efficiency of expert discussion and obtain the consistent high-reliability weight.
4. The calculation process of this evaluation method is simple, the evaluation process is clear, and the evaluation results are intuitive. The obtained correlation degree can not only reflect the stability grade of the anchored slope but also reflect the "distance" between the anchored slope and other stability grades. The feasibility of the evaluation method is verified by an engineering example.

## Data Availability

The datasets generated during and/or analysed during the current study are available from the corresponding author on reasonable request.
